# Radioprotective Effect of Selenium Nanoparticles: A Mini Review

**DOI:** 10.1049/2024/5538107

**Published:** 2024-01-25

**Authors:** Rasool Azmoonfar, Masoud Moslehi, Daryoush Shahbazi-Gahrouei

**Affiliations:** Department of Medical Physics, School of Medicine, Isfahan University of Medical Sciences, Isfahan 81746-73461, Iran

## Abstract

**Materials and Methods:**

This study followed the PRISMA reporting guidelines to present the results. A comprehensive search was performed on electronic databases such as PubMed, Scopus, Web of Sciences, and Science Direct. Initially, 413 articles were retrieved. After removing duplicates and applying specific inclusion and exclusion criteria, 10 articles were finally included in this systematic review.

**Results:**

The reviewed studies showed that selenium nanoparticles had anti-inflammatory and antioxidant properties. They effectively protected the kidneys, liver, and testicles from damage. Furthermore, there was evidence of efficient radioprotection for the organs examined without significant side effects.

**Conclusions:**

This systematic review emphasizes the potential advantages of using selenium nanoparticles to prevent the negative effects of ionizing radiation. Importantly, these protective effects were achieved without causing noticeable side effects. These findings suggest the potential role of selenium nanoparticles as radioprotective agents, offering possible therapeutic applications to reduce the risks related to ionizing radiation exposure in medical imaging and radiotherapy procedures.

## 1. Introduction

Ionizing radiation (IR) is widely used in modern medicine for both diagnosis and treatment. Radiotherapy (RT) is a highly effective and common method for cancer treatment [1–4]. However, this treatment method has a major drawback: it can damage healthy cells and tissues [5]. IR affects cells through direct and indirect mechanisms, causing physiological and pathological changes that may endanger biological tissues [6, 7]. Protecting normal tissues from IR is a vital concern in clinical and environmental radiobiology [8]. The main cause of radiation-induced side effects is the production of free radicals within cells [9].

Over the past decades, many studies have explored the use of radioprotectors to mitigate the detrimental effects of ionizing radiation [9]. Radioprotective agents, or radioprotectors, have been suggested to avoid or reduce IR side effects [10]. The ideal radioprotector provides optimal protection for healthy tissues and organs, while minimizing toxicity and targeting healthy cells rather than the cancer cells [11]. Radioprotectors function through various mechanisms such as antioxidant and anti-inflammatory activities, prevention of DNA damage, elimination of free radicals, and activation of the body's repair mechanisms [12–15].

Selenium, a crucial natural element that participates in important biological processes such as immune function, thyroid hormone regulation, and antioxidant defenses, has shown its ability to protect cells from the oxidative damage and lower inflammation [16–18].

Nanoparticles have various applications in medicine, such as enhancing radiation sensitivity and protection, improving diagnostic imaging, treating cancer, and delivering drugs [19–22]. Selenium nanoparticles (SeNPs) are of particular interest for their possible protective properties [23, 24]. SeNPs have very small dimensions, ranging from 1 to 500 nm [25]. Many studies have demonstrated the potential of SeNPs to shield healthy cells and tissues from the harmful effects of radiation and to increase the effectiveness of radiation therapy. Moreover, selenium nanoparticles are biocompatible and have low toxicity, which makes them suitable candidates for radioprotection [26].

## 2. Materials and Methods

### 2.1. Search Strategy

The Statement of Priority Reporting Items for Systematic Reviews and Meta-Analyses (PRISMA) was followed to report this systematic review [27]. In March 2023, a comprehensive computerized search of relevant English-language studies was conducted. The databases such as PubMed, Scopus, Science Direct, and Web of Science were used and they did not limit the publication years. The terms “radiation,” “Selenium,” and any of the following: “nano-particle,” “nanoparticle,” or “nano particle” were searched. The references of the selected studies to find more related publications were also manually checked.

### 2.2. Inclusion Criteria

Certain criteria were applied to select the articles for this review, as follows:

- The studies had to examine the radioprotective effects of SeNPs and be written in English.
- The studies had to use ionizing radiation.
- The studies had to contain the keywords we specified earlier and provide sufficient information.
- The studies could be of any publication type, such as clinical, *in vivo*, or *in vitro* research.
- These criteria ensured that the articles we chose for our review were relevant and met the necessary standards.

### 2.3. Exclusion Criteria

This review excluded studies based on these criteria:

- Studies that did not relate to SeNPs.
- Research that used other kinds of radiation, such as RF-EMF, ultraviolet (UV), fluorescence, or cosmic radiation.
- Studies that assessed the effects of SeNPs with chemotherapy instead of radiation therapy.
- Review articles, case reports, conference abstracts, simulation studies, letters to editors, unpublished data, editorials, articles without full texts, and articles that were not in English.

To maintain the focus and relevance of this research, the mentioned criteria was used to select articles that explicitly discussed the use of SeNPs for radiation therapy.

### 2.4. Study Selection

The articles from electronic and manual searches were imported into Endnote software for organization and duplicate articles from the dataset were removed. Only the studies that met the predefined inclusion criteria for further analysis were chosen.

### 2.5. Data Extraction

The following details from each article were extracted after two researchers independently reviewed them: the first author's name, publication year, subject, organ or tissue of interest, radiation type and dose, dosage, and findings. Also, the key findings were synthesized and analyzed.

## 3. Results

To assess the radioprotective effect of SeNPs and synthesize the existing evidence, a systematic review was conducted. The established guidelines to perform a systematic literature search were followed. Relevant studies that investigated the radioprotective effect of SeNPs were included. The results from eligible studies were pooled after conducting quality assessment and data extraction.

### 3.1. Literature Search and Screening

**Figure 1:**
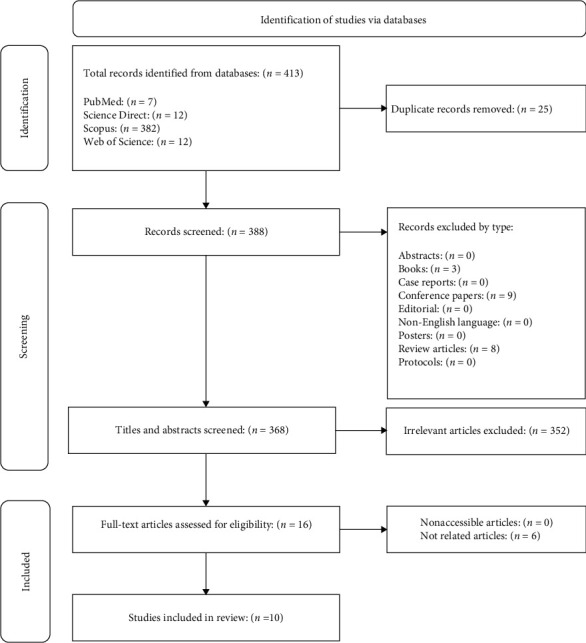
The process of selecting studies and the results.

Figure 1 illustrates the process of selecting studies and the results of this work. Number of 413 articles was collected from the electronic databases mentioned through an extensive search. These articles were published from 2013 to 2022. Duplicate articles (*n* = 25) were excluded and evaluated the remaining articles (*n* = 388) by their article type, title, and abstract. Then, 352 irrelevant articles were excluded. Finally, 16 articles were examined in their full text and included 10 articles in the systematic review based on inclusion and exclusion criteria.

### 3.2. Study Characteristics

The characteristics of the studies included in our analysis are summarized in Table 1. Eight studies used rats and mice as *in vivo* experimental models, while two studies used lymphocyte and CHO cells as *in vitro* models. Gamma rays were the radiation type in seven studies, and X-rays were the radiation type in three studies. The radiation doses ranged from 0.04 to 8 Gy.

The *in vivo* studies administered SeNPs orally in five studies and intraperitoneally (IP) in three studies. The routes of administration depended on the experimental setup and objectives of each study.

### 3.3. Various Methods of SeNPs Synthesis and Advantages in the Radioprotective Application

Elemental selenium in the form of nanoscale particles (SeNPs) has drawn the attention of many researchers for its biocompatibility, bioavailability, and low toxicity. Various synthesis methods, such as physical, chemical, and biological methods, can be used to produce SeNPs, each with its own merits and drawbacks. Physical methods, such as laser ablation and gamma irradiation, can yield SeNPs with high purity and uniform size, but they are costly and require advanced equipment. Chemical methods, such as reduction and solvothermal synthesis, can generate SeNPs with different shapes and sizes, but they may use toxic reagents and produce harmful byproducts. Biological methods, such as using microorganisms, plants, and biomolecules, can create SeNPs with environmentally friendly and economical approaches, but they may have low yield and stability issues [37–39].

The size of SeNPs is a key factor that affects their physicochemical and biological properties, such as solubility, stability, surface area, cellular uptake, and biodistribution. The size and the shape of SeNPs depend on the synthesis method used, and they can range from 1 to 500 nm. Smaller SeNPs have higher surface-to-volume ratios and more reactive sites, which may improve their antioxidant and radioprotective activities [38, 40–42]. However, the optimal size range of SeNPs for different biomedical applications remains to be determined. Selenium nanoparticles have potential applications in the biomedical field, such as in the treatment of infections, cancer, diabetes, and inflammation, as well as in the prevention of oxidative stress. Selenium nanoparticles have demonstrated promising applications in radiation protection, as they can act as antioxidants, anti-inflammatory agents, and chemopreventive agents. Selenium nanoparticles can scavenge reactive oxygen species (ROS) produced by ionizing radiation, protect normal cells from DNA damage and apoptosis, and increase the radiosensitivity of tumor cells. Selenium nanoparticles can also modulate the immune system and prevent the inflammation and fibrosis induced by radiation exposure [31, 34, 43]. Therefore, SeNPs can be used as potential radioprotectors for the prevention and treatment of radiation-induced injuries.

### 3.4. SeNPs Dosage

The optimal dose of SeNPs for radioprotection has been investigated in various studies. The studies that mainly used a dose of 0.5 mg/kg body weight of SeNPs to assess its radioprotective effects were included for analysis. This dose of SeNPs was effective in preventing kidney and liver damage caused by IR. This dose also showed potential in reducing the adverse effects of IR on these organs [26, 29, 32].

### 3.5. Radiation Damages and Radioprotective Effect of SeNPs

**Figure 2:**
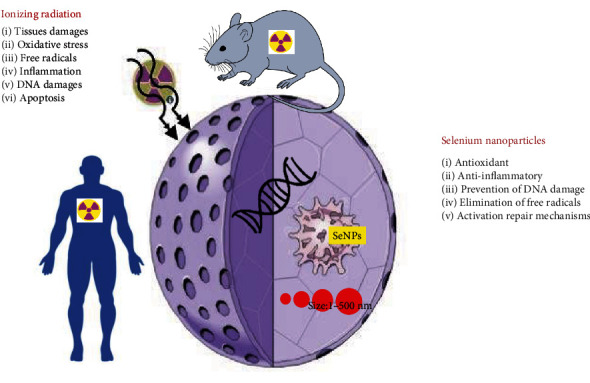
Selenium nanoparticles attenuate damage induced by ionizing radiation in biological systems.

IR exposure at different doses can harm various organs and cells, both *in vitro* and *in vivo* (Figure 2). Pretreatment or posttreatment with SeNPs can reduce the harmful effects of radiation on the bone marrow, liver, kidney, and testes. Many studies showed that SeNPs can protect against radiation-induced injury and inflammation in animals.

The study by Yazdi et al. [28] found that a single dose of 8 Gy caused various toxicities that affected the bone marrow and lowered the WBC counts. These toxicities are serious side effects of radiation exposure. SeNPs enhanced bone marrow function and raised the number of lymphocytes and neutrophils, indicating its potential to prevent radiation-induced bone marrow damage. Moreover, SeNPs accumulated in the liver and helped a faster recovery [28].

Fahmy et al. [29] found that Cisplatin and *γ*-irradiation increased renin and IL6 levels in the blood and reduced antioxidant activity. These biomarker changes revealed the mechanism of liver damage. The researchers also showed that SeNPs and fish oil could lower liver damage from radiation and cisplatin. This implies that SeNPs could protect the liver [29].

The report showed that nicotine alone or with IR harmed the kidneys. This was clear from the rise in some markers of kidney function in the blood, along with oxidative and inflammatory changes. Zahran et al. [30] showed that SeNPs prevented kidney damage, lowered inflammation, and removed free radicals in rats exposed to whole-body irradiation. These findings emphasize the antioxidant and anti-inflammatory features of SeNPs, which help to protect the kidneys [30].

In addition, El-Ghazaly et al. [31] showed that SeNPs treatment decreased inflammation and edema in rats exposed to radiation, suggesting that SeNPs have anti-inflammatory and antioxidant effects by suppressing proinflammatory genes. Karami et al. [26] evaluated the protective role of SeNPs against nephropathy induced by IR in mice. They reported that SeNPs, which have strong antioxidant properties, could prevent nephropathy effectively [26]. Saif-Elnasr et al. [32] demonstrated that fish oil and/or SeNPs pretreatment lowered kidney damage in rats caused by cisplatin and radiation. The pretreatment also enhanced renal antioxidant status, reduced Casp-3 and COX-2 levels, and decreased various renal indicators [32]. Furthermore, SeNPs exhibited antioxidant activity, alleviating liver injury, and lowering enzyme levels related to liver function in rats subjected to gamma-ray radiation [33]. Jafarpour et al. [34] explored the effect of melatonin and SeNPs combination and observed that it reduced DNA double-strand breaks in peripheral lymphocytes exposed to ionizing radiation, implying a possible protective effect.

It is essential to acknowledge that more research is needed to fully elucidate the mechanisms behind these radioprotective effects and to identify the best dosages and treatment regimens. Thorough testing and assessment are vital before applying these results to the clinical or practical settings [35, 36]. In summary, the studies reviewed in this collection highlight the potential of SeNPs in mitigating the adverse effects of radiation on various organs and tissues. However, further research is required to confirm these results and determine the most optimal therapeutic approaches. These useful insights enhance the growing knowledge of SeNPs as possible radioprotective agents, opening up new avenues for the future research in this area.

## 4. Discussion

Radiation therapy aims to destroy cancer cells, but it may also affect the surrounding healthy tissues. Healthcare professionals have to weigh the pros and cons of radiation therapy, considering how well it kills cancer cells and how much damage it causes to nearby tissues [44]. Infrared exposure can harm the cellular components of living organisms, and the extent of the harm varies with the radiation dose and the organ affected [45–47]. Radiobiology is greatly concerned with safeguarding normal tissues from the adverse effects of IR [8].

Radioprotectors can help to mitigate radiation hazards. Nanoparticles are widely used in various medical fields, including radiation protection [48–52]. This study systematically reviewed the role of SeNPs as radioprotectors against IR-induced damage. Selenium is an essential element for the body's functions. SeNPs have lower toxicity and better radioprotective effects than other selenium compounds in medicine, suggesting their potential for future biomedical applications [53–57].

SeNPs can fight cancer when used with immunotherapy, chemotherapy, or RT [25, 58, 59]. Moreover, studies have revealed that SeNPs with natural ingredients such as GSE can benefit diabetic patients who receive RT [60].

The studies in this review have shown that SeNPs can protect animals, cultured cells, and various tissues from gamma rays and X-rays (Table 1). In the bone marrow, SeNPs improved bone marrow function and raised lymphocyte and neutrophil counts, suggesting that they can prevent radiation-induced bone marrow suppression [28]. Likewise, studies on the liver showed that SeNPs reduced liver damage from IR and chemical exposure, demonstrating their protective effect on hepatic tissue [29].

Studies on the kidney showed that SeNPs prevented kidney damage, lowered inflammation, and eliminated free radicals, indicating that their antioxidant and anti-inflammatory properties enhanced the radioprotective effects on renal tissue [30]. SeNPs also reduced inflammation and edema in irradiated rats, revealing their potential anti-inflammatory activities through the suppression of proinflammatory genes and antioxidant effects [31]. SeNPs protected against nephropathy from IR exposure due to their strong antioxidant properties [26]. Moreover, SeNPs pretreatment decreased kidney damage from radiation and chemical exposure, boosted renal antioxidant capacity, and lowered the levels of renal markers, confirming their potential as radioprotective agents [32].

This review presents strong evidence for the protective role of SeNPs against radiation-induced harm in various organs and tissues. The results indicate that SeNPs could be used to prevent or reduce the damage and inflammation caused by radiation exposure. The antioxidant activity of SeNPs, which eliminates free radicals and lowers oxidative stress, may explain the observed protection. Moreover, the anti-inflammatory effects and regulation of inflammatory genes by SeNPs also enhance the overall protection against radiation damage. However, it should be noted that the findings of this review are not conclusive, and more research is needed to overcome some limitations. The studies included in this review differed in their methods, doses, and treatments, which could affect the outcomes. Also, the studies mainly used animal models, and more human studies are required to confirm these results.

## 5. Conclusions and Future Perspectives

This review article investigated the protective role of SeNPs against radiation damage. By analyzing relevant studies, it showed that SeNPs have considerable promise in reducing the negative effects of radiation on different tissues. Histopathological assessments have repeatedly shown the ability of SeNPs to lessen pathological alterations and maintain tissue structure. These results emphasize the radioprotective features of SeNPs and may have useful implications for future research and clinical use. More studies are needed to fine-tune the dose and delivery of SeNPs, enabling the development of efficient methods to improve their protective effects. By utilizing the potential of SeNPs, the patient outcomes and lower the harmful impacts of radiation therapy on healthy tissues may be enhanced.

## Figures and Tables

**Table 1:** Summary of articles included in this review.

First author	Organ (or tissue) of interest	Radiation type and dose	SeNPs dose/concentration	Time for outcome assessment	Main outcomes
Yazdi et al. [28]	Blood, heart, brain, liver, spleen, and lung	X-ray, 2, 4, and 8 Gy, whole body irradiation	100 *μ*g/day, oral 30 days	10, 20, and 30 days	1. SeNPs help to restore bone marrow function and significantly increase the numbers of lymphocytes and neutrophils in mice that have suffered from bone marrow suppression in irradiated mice 2. Deposition of SeNPs in the liver 3. Better recovery, especially in groups exposed to radiation with doses of 2 and 4 Gy
Fahmy et al. [29]	Blood, liver tissue (renin, IL6, PON-1, and GPx activity)	*γ*-Ray, 0.7 Gy, whole body irradiation	0.5 mg/kg, oral	24 hr	1. A combination of SeNPs and fish oil has the potential to mitigate the harm the liver caused by exposure to IR and cisplatin
Zahran et al. [30]	Blood and kidney tissue	*γ*-Ray, 0.5 Gy, whole body irradiation	0.1 mg/kg, oral	24 hr	SeNPs effectively prevent kidney damage and have the ability to scavenge free radicals and protect against nicotine toxicity by reducing inflammation through antioxidant activity
El-Ghazaly et al. [31]	Paw edema	*γ*-Ray, 6 Gy	2.55 mg/kg, oral	24 hr	1. Nano-Se was able to reduce paw edema in inflamed and irradiated rats by 35% and 47%, respectively 2. Anti-inflammatory activity may occur through the downregulation of pro-inflammatory genes and/or in addition to their antioxidant activity
Karami et al. [26]	Blood and kidney tissue	*γ*-Ray, 2 and 8 Gy	0.5 mg/kg, IP	48 hr	SeNPs, which are a newly recognized powerful antioxidant agent, have the ability to protect against nephropathy caused by IR exposure
Saif-Elnasr et al. [32]	Blood and kidney tissue	*γ*-Ray, 0.7 Gy	0.5 mg/kg, oral/12 day	24 hr	Pretreatment with fish oil and/or SeNPs: 1. Prevention of kidney damage caused by cisplatin and radiation 2. Reduced CP and *γ*-radiation-induced high levels in renal Casp-3 and COX-2 activities 3. Ameliorated the renal levels of trace elements, urea, creatinine, and TNF-a 4. Increased renal TAC levels
Sohrabi et al. [33]	Blood and liver tissue	*γ*-Ray, 2 and 8 Gy	0.10 mg/kg, IP	72 hr	1. Decreased liver damage, antioxidant effect 2. Reducing the response recorded in enzyme activity of AST, ALT, ALP, and GGT
Jafarpour et al. [34]	Lymphocyte	*γ*-Ray, 20 *µ*Ci 40 mGy	0.025 mg/mL	2 hr	The levels of DSBs caused by IR in peripheral lymphocytes decreased by utilizing melatonin and SeNPs
Hasanzadeh et al. [35]	CHO cells	X-ray, 0.5, 1, and 2 Gy	0.21625, 0.4325, 0.865, 1.73 ppm	24 hr	The process of creating selenium nanoparticles in rosemary extract did not have a combined protective effect against IR on CHO cells
Ehghaghi et al. [36]	Testis	X-ray, 2 Gy	0.2 mg/kg, oral	NI, not informed	SeNPs and probiotics reduce testicular damage and improve antioxidant status in male mice

## Data Availability

The data presented in this study are available on request from the corresponding author.

## References

[1] Khodamoradi E., Azmoonfar R., Mohammadi M. (2020). Transfer of radio-adaptation via serum: a preliminary report. *Iranian Journal of Medical Physics*.

[2] Musa A. E., Omyan G., Esmaely F., Shabeeb D. (2019). Radioprotective effect of hesperidin: a systematic review. *Medicina*.

[3] Ford E. C., Terezakis S. (2010). How safe is safe? Risk in radiotherapy. *International Journal of Radiation Oncology, Biology, Physics*.

[4] Mortezaee K., Parwaie W., Motevaseli E. (2019). Targets for improving tumor response to radiotherapy. *International Immunopharmacology*.

[5] Azmoonfar R., Amini P., Saffar H. (2020). Celecoxib a selective COX-2 inhibitor mitigates fibrosis but not pneumonitis following lung irradiation: a histopathological study. *Current Drug Therapy*.

[6] Liu L., Liang Z., Ma S., Li L., Liu X. (2023). Radioprotective countermeasures for radiation injury (Review). *Molecular Medicine Reports*.

[7] Khurana H., Hazari P. P., Mishra A. K. (2019). Radioprotective efficacy of GSH based peptidomimetic complex of manganese against radiation induced damage: DT(GS)2Mn(II). *Free Radical Biology and Medicine*.

[8] Musa A. E., Shabeeb D., Okoro N. O. E., Agbele A. T. (2020). Radiation protection by Ex-RAD: a systematic review. *Environmental Science and Pollution Research*.

[9] Rezaeyan A., Haddadi G. H., Hosseinzadeh M., Moradi M., Najafi M. (2016). Radioprotective effects of hesperidin on oxidative damages and histopathological changes induced by X-irradiation in rats heart tissue. *Journal of Medical Physics/Association of Medical Physicists of India*.

[10] Azmoonfar R., Khosravi H., Rafieemehr H. (2023). Radioprotective effect of malva sylvestris L. against radiation-induced liver, kidney and intestine damages in rat: a histopathological study. *Biochemistry and Biophysics Reports*.

[11] Citrin D., Cotrim A. P., Hyodo F., Baum B. J., Krishna M. C., Mitchell J. B. (2010). Radioprotectors and mitigators of radiation-induced normal tissue injury. *The Oncologist*.

[12] Kim J. H., Kim K. M., Jung M. H. (2016). Protective effects of alpha lipoic acid on radiation-induced salivary gland injury in rats. *Oncotarget*.

[13] Nadi S., Banaei A., Mozdarani H., Monfared A. S., Ataei G., Abedi-Firouzjah R. (2020). Evaluating the radioprotective effect of arbutin on mice exposed to megavoltage X-rays based on hematological parameters and lymphocytes micronucleus assay. *International Journal of Radiation Research*.

[14] Hosseinimehr S. J. (2007). Trends in the development of radioprotective agents. *Drug Discovery Today*.

[15] Azmoonfar R., Amini P., Saffar H. (2018). Metformin protects against radiation-induced pneumonitis and fibrosis and attenuates upregulation of dual oxidase genes expression. *Advanced Pharmaceutical Bulletin*.

[16] Jiang W., He S., Su D., Ye M., Zeng Q., Yuan Y. (2022). Synthesis, characterization of tuna polypeptide selenium nanoparticle, and its immunomodulatory and antioxidant effects in vivo. *Food Chemistry*.

[17] Yazdi M. H., Mahdavi M., Kheradmand E., Shahverdi A. R. (2012). The preventive oral supplementation of a selenium nanoparticle-enriched probiotic increases the immune response and lifespan of 4T1 breast cancer bearing mice. *Arzneimittelforschung*.

[18] Gao F., Yuan Q., Gao L. (2014). Cytotoxicity and therapeutic effect of irinotecan combined with selenium nanoparticles. *Biomaterials*.

[19] Aminolroayaei F., Shahbazi-Gahrouei S., Khorasani A., Shahbazi-Gahrouei D. (2023). A review of imaging methods and recent nanoparticles for breast cancer diagnosis. *Information*.

[20] Shahbazi-Gahrouei D., Choghazardi Y., Kazemzadeh A., Naseri P., Shahbazi-Gahrouei S. (2023). A review of bismuth-based nanoparticles and their applications in radiosensitising and dose enhancement for cancer radiation therapy. *IET Nanobiotechnology*.

[21] Khorasani A., Shahbazi-Gahrouei D., Safari A. (2023). Recent metal nanotheranostics for cancer diagnosis and therapy: a review. *Diagnostics*.

[22] Aminolroayaei F., Shahbazi-Gahrouei D., Shahbazi-Gahrouei S., Rasouli N. (2021). Recent nanotheranostics applications for cancer therapy and diagnosis: a review. *IET Nanobiotechnology*.

[23] Bhattacharjee A., Basu A., Ghosh P., Biswas J., Bhattacharya S. (2014). Protective effect of selenium nanoparticle against cyclophosphamide induced hepatotoxicity and genotoxicity in swiss albino mice. *Journal of Biomaterials Applications*.

[24] Chen Y., Qiao L., Song X. (2021). Protective effects of selenium nanoparticle-enriched lactococcus lactis NZ9000 against enterotoxigenic escherichia coli K88-induced intestinal barrier damage in mice. *Applied and Environmental Microbiology*.

[25] Chen F., Zhang X. H., Hu X. D., Liu P. D., Zhang H. Q. (2018). The effects of combined selenium nanoparticles and radiation therapy on breast cancer cells *in vitro*. *Artificial Cells, Nanomedicine, and Biotechnology*.

[26] Karami M., Asri-Rezaei S., Dormanesh B., Nazarizadeh A. (2018). Comparative study of radioprotective effects of selenium nanoparticles and sodium selenite in irradiation-induced nephropathy of mice model. *International Journal of Radiation Biology*.

[27] Moher D., Liberati A., Tetzlaff J., Altman D. G. (2009). Preferred reporting items for systematic reviews and meta-analyses: the PRISMA statement. *Annals of Internal Medicine*.

[28] Yazdi M. H., Masoudifar M., Varastehmoradi B. (2013). Effect of oral supplementation of biogenic selenium nanoparticles on white blood cell profile of BALB/c mice and mice exposed to X-ray radiation. *Avicenna Journal of Medical Biotechnology*.

[29] Fahmy H. A., Abd El Azim A. S., Gharib O. A. (2016). Protective Effects of omega-3 fatty acids and/or nanoselenium on cisplatin and ionizing radiation induced liver toxicity in rats. *Indian Journal of Pharmaceutical Education and Research*.

[30] Zahran W. E., Elsonbaty S. M., Moawed F. S. M. (2017). Selenium nanoparticles with low-level ionizing radiation exposure ameliorate nicotine-induced inflammatory impairment in rat kidney. *Environmental Science and Pollution Research*.

[31] El-Ghazaly M. A., Fadel N., Rashed E., El-Batal A., Kenawy S. A. (2017). Anti-inflammatory effect of selenium nanoparticles on the inflammation induced in irradiated rats. *Canadian Journal of Physiology and Pharmacology*.

[32] Saif-Elnasr M., Abdel-Aziz N., El-Batal A. I. (2019). Ameliorative effect of selenium nanoparticles and fish oil on cisplatin and gamma irradiation-induced nephrotoxicity in male albino rats. *Drug and Chemical Toxicology*.

[33] Sohrabi A., Tehrani A. A., Asri-Rezaei S., Zeinali A., Norouzi M. (2020). Histopathological assessment of protective effects of selenium nanoparticles on rat hepatocytes exposed to gamma radiation. *Veterinary Research Forum*.

[34] Jafarpour S. M., Shekarchi B., Bagheri H., Farhood B. (2021). The radioprotective effects of melatonin and nanoselenium on DNA double-strand breaks in peripheral lymphocytes caused by I-131. *Indian Journal of Nuclear Medicine*.

[35] Hasanzadeh M., Toossi M. T. B., Vaziri-Nezamdoost F., Khademi S., Darroudi M., Azimian H. (2022). Comparison of radioprotective effects of colloidal synthesis of selenium nanoparticles in aqueous rosemary extract and rosemary in chinese hamster ovary (CHO) cells. *Journal of Nanostructures*.

[36] Ehghaghi A., Zokaei E., Modarressi M. H. (2022). Antioxidant and anti-apoptotic effects of selenium nanoparticles and *Lactobacillus casei* on mice testis after X-ray. *Andrologia*.

[37] Pyrzynska K., Sentkowska A. (2021). Biosynthesis of selenium nanoparticles using plant extracts. *Journal of Nanostructure in Chemistry*.

[38] Bisht N., Phalswal P., Khanna P. K. (2022). Selenium nanoparticles: a review on synthesis and biomedical applications. *Materials Advances*.

[39] Pereira A. G., Gerolis L. G. L., Gonçalves L. S., Moreira L. M. C., Gastelois P. L., Neves M. J. (2022). Radiolytic synthesis and characterization of selenium nanoparticles: comparative biosafety evaluation with selenite and ionizing radiation. *World Journal of Microbiology and Biotechnology*.

[40] Husen A., Siddiqi K. S. (2014). Plants and microbes assisted selenium nanoparticles: characterization and application. *Journal of Nanobiotechnology*.

[41] Wadhwani S. A., Shedbalkar U. U., Singh R., Chopade B. A. (2016). Biogenic selenium nanoparticles: current status and future prospects. *Applied Microbiology and Biotechnology*.

[42] Nayak V., Singh K. R. B., Singh A. K., Singh R. P. (2021). Potentialities of selenium nanoparticles in biomedical science. *New Journal of Chemistry*.

[43] Gudkov S. V., Gao M., Simakin A. V. (2023). Laser ablation-generated crystalline selenium nanoparticles prevent damage of DNA and proteins induced by reactive oxygen species and protect mice against injuries caused by radiation-induced oxidative stress. *Materials*.

[44] Rodemann H. P., Blaese M. A. (2007). Responses of normal cells to ionizing radiation. *Seminars in Radiation Oncology*.

[45] Singh V., Gupta D., Arora R. (2015). NF-*κ*B as a key player in regulation of cellular radiation responses and identification of radiation countermeasures. *Discoveries*.

[46] Yahyapour R., Shabeeb D., Cheki M. (2018). Radiation protection and mitigation by natural antioxidants and flavonoids: implications to radiotherapy and radiation disasters. *Current Molecular Pharmacology*.

[47] Manda K., Glasow A., Paape D., Hildebrandt G. (2012). Effects of ionizing radiation on the immune system with special emphasis on the interaction of dendritic and T cells. *Frontiers in Oncology*.

[48] Long W., Wang J., Yang J. (2019). Naturally-derived PHA-L protein nanoparticle as a radioprotector through activation of toll-like receptor 5. *Journal of Biomedical Nanotechnology*.

[49] Zal Z., Ghasemi A., Azizi S., Asgarian-Omran H., Montazeri A., Hosseinimehr S. J. (2018). Radioprotective effect of cerium oxide nanoparticles against genotoxicity induced by ionizing radiation on human lymphocytes. *Current Radiopharmaceuticals*.

[50] Feliciano C. P., Tsuboi K., Suzuki K., Kimura H., Nagasaki Y. (2017). Long-term bioavailability of redox nanoparticles effectively reduces organ dysfunctions and death in whole-body irradiated mice. *Biomaterials*.

[51] Kadivar F., Haddadi G., Mosleh-Shirazi M. A., Khajeh F., Tavasoli A. (2020). Protection effect of cerium oxide nanoparticles against radiation-induced acute lung injuries in rats. *Reports of Practical Oncology & Radiotherapy*.

[52] Xie J., Wang N., Dong X. (2019). Graphdiyne nanoparticles with high free radical scavenging activity for radiation protection. *ACS Applied Materials & Interfaces*.

[53] Agarwal R., Behari J. R. (2007). Effect of selenium pretreatment in chronic mercury intoxication in rats. *Bulletin of Environmental Contamination and Toxicology*.

[54] Zhai X., Zhang C., Zhao G., Stoll S., Ren F., Leng X. (2017). Antioxidant capacities of the selenium nanoparticles stabilized by chitosan. *Journal of Nanobiotechnology*.

[55] Farhood B., Mortezaee K., Motevaseli E. (2019). Selenium as an adjuvant for modification of radiation response. *Journal of Cellular Biochemistry*.

[56] Ferro C., Florindo H. F., Santos H. A. (2021). Selenium nanoparticles for biomedical applications: from development and characterization to therapeutics. *Advanced Healthcare Materials*.

[57] Wang H., Zhang J., Hai-Qing Y. (2007). Effect of elemental selenium at nano size (Nano-Se) with lower toxicity on the anticancer effect of cisplatin. *Acta Nutrimenta Sinica*.

[58] Tian J., Wei X., Zhang W., Xu A. (2020). Effects of selenium nanoparticles combined with radiotherapy on lung cancer cells. *Frontiers in Bioengineering and Biotechnology*.

[59] Gao S., Li T., Guo Y., Sun C., Xianyu B., Xu H. (2020). Selenium-containing nanoparticles combine the NK cells mediated immunotherapy with radiotherapy and chemotherapy. *Advanced Materials*.

[60] Abdelaleem R. M. A., Abdel Hameed H., Askar M., Hassan S. H. M., El-Batal A. I. (2016). Modulatory role of selenium nanoparticles and grape seed extract mixture on oxidative stress biomarkers in diabetic irradiated rats. *Indian Journal of Pharmaceutical Education and Research*.

